# An evaluation of serum blood parameters and amyloid-A levels in women with hyperemesis gravidarum; A prospective observational study

**DOI:** 10.1097/MD.0000000000039695

**Published:** 2024-09-20

**Authors:** Durmus Onder, Meryem Busra Birsen, Derya Erturk, Ahmet Ilker Eryilmaz, Ozgur Ozdemir, Guzin Aykal, Zeynep Ozturk Inal

**Affiliations:** a Antalya Training and Research Hospital, Department of Obstetrics and Gynecology, Antalya, Turkey; b Konya City Hospital, Department of Obstetrics and Gynecology, Konya, Turkey.

**Keywords:** Biomarker, hyperemesis gravidarum, serum amyloid A

## Abstract

This study aimed to investigate whether serum amyloid A (AA) level can be used as a biomarker in women with hyperemesis gravidarum (HEG). This prospective observational study was conducted at the Antalya Training and Research Hospital Gynecology and Obstetrics Clinic, Türkiye, between July and December 2023. Forty women diagnosed with HEG and 40 healthy women were included. No statistically significant differences were observed between the groups in terms of sociodemographic data such as age, body mass index, family history, educational status, economic level, place of residence, occupation, smoking and alcohol use, or drug habits. However, obstetric characteristics such as number of miscarriages, number of dilatation curettages, and gestational age and laboratory values including complete blood count, hematocrit, leukocyte, neutrophil, lymphocyte, platelet, free T4, albumin, alanine aminotransferase, aspartate aminotransferase, urea, creatinine, hs-C-reactive protein, and sodium (*P* > .05) all differed significantly. In addition, significant differences were observed between the HEG and healthy groups in terms of numbers of gravidities (2 [1–3] vs 1 [0–1], respectively, *P* < .001), numbers of parities (1 [0–1] vs 1 [0–1], *P* < .001), numbers of living children (1 [0–2] vs 1 [0–1], *P* < .001), presenting complaints (nausea 0 [0%], nausea + vomiting 0 [0%], none 40 [100.0%] vs nausea 27 [67.5%], nausea + vomiting 13 [32.5%], none 0 [0%], *P* < .001), serum thyroid-stimulating hormone (1.16 ± 0.56 vs 1.81 ± 0.624, *P* = .004), potassium (4.1 ± 0.7 vs 3.8 ± 0.2, *P* = .001), and AA values (7.29 ± 2.61 vs 10.74 ± 3.04, *P* < .001). At receiver operating characteristic analysis, the area under the curve (AUC: 0.881) was statistically significant for serum AA (*P*: <.001), with a cutoff value of ≥ 8.79 ([95% confidence interval] 0.743–0.919, sensitivity 87.4%, specificity 80.2%). The positive predictive value of serum AA was 81.1% and the negative predictive value was 80.4%. The study results showed that serum AA can be used as a diagnostic biomarker in HEG. Prospective studies involving more participants are now required to confirm our results.

## 1. Introduction

Hyperemesis gravidarum (HEG) is a severe condition marked by extreme nausea and vomiting during pregnancy. It involves persistent vomiting that leads to ketonuria and/or ketonemia, resulting in weight loss and a reduction in body volume exceeding 5% of pre-pregnancy weight.^[1–5]^ Affecting approximately 2% of pregnancies in the United States, HEG can severely impact a woman’s ability to perform daily tasks, and contribute to anxiety and depression. In some cases, it may even prompt patients to terminate the pregnancy or avoid planning future pregnancies.^[1–5]^ Vomiting in severe HEG may cause dehydration, fluid-electrolyte imbalance, hyponatremia, hypokalemia, and hemoconcentration.^[6,7]^ Use of the Rhodes and the Pregnancy Unique Quantification of Emesis and Nausea (PUQE-24) classification system is recommended when evaluating the severity of nausea and vomiting in HEG.^[8,9]^ Prognosis in HEG is generally good, as a result of advances in medical treatment options. However, the condition can cause maternal and fetal problems such as spontaneous abortion, prematurity, congenital malformation, fetal growth restriction, preeclampsia, and low birth weight.^[6,8]^ Maternal complications in severe HEG include weight loss, dehydration, acidosis due to malnutrition, metabolic alkalosis, hypokalemia, muscle weakness, and bleeding disorders due to vitamin K deficiency.^[7,9]^

Amyloid A (AA) is a molecule synthesized by the liver in acute inflammatory events. Its levels can rise up to 1000 times above the normal serum value, and serum levels remain higher than normal in chronic inflammatory diseases, although it is unclear whether it plays a role in the etiopathogenesis of these diseases.^[10]^ Increased serum levels are a poor prognostic criterion in atherosclerotic and cardiovascular diseases, as well as cancer.^[11]^ The great majority (95%) of AA is associated with high-density lipoprotein, and in severe acute inflammatory events it becomes the main apolipoprotein on high-density lipoprotein and induces cytokine production through immune mechanisms.^[12]^ Serum AA levels rise in diseases such as colorectal carcinoma, ovarian cancer, uterine carcinoma, glioblastoma multiforme, pancreatic adenocarcinoma, and neonatal encephalopathy.^[13]^

Metabolic and endocrine factors (human chorionic gonadotropin [hCG], progesterone, thyroid hormones, and prostaglandin E2), genetic and immunological factors, irregular and unbalanced eating habits, vestibular system disorders, gastrointestinal motility dysfunction, and inflammations have been implicated in the etiopathogenesis of HEG, a known multifactorial disease.^[4,5]^ Since serum AA levels increase in inflammation and inflammation plays a role in the etiopathogenesis of HEG, this study has been carried out in order to evaluate serum blood parameters and serum AA levels in pregnant women with HEG.

## 2. Material and methods

This prospective, observational study was conducted at the Antalya Training and Research Hospital Gynecology and Obstetrics Clinic, Türkiye, between April and October 2023. Eighty pregnant women diagnosed with HEG and 40 healthy pregnant women with no complaints, whose measurements were compatible with similar gestational weeks, were included. Local ethics committee approval was obtained for the study (Antalya Training and Research Hospital Ethics Committee, reference number 2023-112), which was conducted in line with the principles of the Declaration of Helsinki. The study was explained to all the participants, after which the informed consent form was read. Consent was finally obtained by means of a wet signature.

The inclusion criteria were age between 18 and 40 years, single live pregnancy, and onset of symptoms before the 20th week of pregnancy. Exclusion criteria were a history of chronic disease (heart failure, thyroid function test disorder, diabetes mellitus, hematological diseases, hypertension, history of thrombophilia, hepatic and renal disease, autoimmune diseases, malignant disease, or chronic inflammatory disease), multiple pregnancies, pregnancies under the age of 18 or over the age of 40, and missing information in the hospital automation database. HEG has been defined as a complication that can cause weight loss, electrolyte imbalance, and renal dysfunction accompanied by persistent nausea and vomiting, and that can have severe effects on the fetus. An ultrasound device (Hitachi HIVISION Avius, Hiatchi, Japan) was used to visualize the pregnancy, the images being evaluated by a single clinician (DO).

Age at the time of presentation, body mass index (BMI), family history, educational status (illiterate, primary school, high school, or university), economic status (low, medium, or high), place of residence (village, district, or province), occupation (housewife, office worker, or manual worker), sociodemographic data such as smoking status, alcohol use, drug habits, numbers of pregnancies, births, living children, and miscarriages, dilatation and curettage, gestational week, presentation complaints (vaginal bleeding, vaginal bleeding + groin pain, or none), obstetric data such as CRL, and laboratory data such as complete blood count, hematocrit, leukocyte, neutrophil, lymphocyte, platelet, free thyroxine (T4), high-sensitivity C-reactive protein (hs-CRP), and AA values for all participants were recorded in the database.

Parameters such as complete blood count, hematocrit, leukocyte, neutrophil, lymphocyte, platelet, free T4, and hs-CRP values measured from blood samples collected at the time of initial admission were calculated using a Coulter LH – 750 device (Beckman Coulter Brea, Brea). The neutrophil – lymphocyte ratio (NLR) was calculated by dividing the absolute neutrophil number by the lymphocyte number, the platelet-lymphocyte ratio (PLR) by dividing the absolute neutrophil number by the lymphocyte number, and BMI by dividing the square of the individual’s height in meters by her body weight.

### 2.1. Serum AA measurement

Venous blood samples were collected into 5.0 mL gel clot activator tubes (Vacusera, Izmir, Türkiye) between 8.00 and 10.00 am after 12-hour fasting. The samples were inverted 5 to 6 times, left at room temperature for 30 minutes to clot, and then centrifuged at 4000 rpm at 6°C for 10 minutes for the serum AA test. The resulting sera were separated into Eppendorf tubes and stored at −80°C in a refrigerator until use. Serum AA was measured using an SEA885Hu enzyme-linked immunosorbent assay (ELISA) kit (Wuhan USCN Business Co. Ltd., Wuhan Economic & Technological Development Zone, China) on an ETIMAX 3000 device (DiaSorin, Saluggia, Italy).

The ELISA method was employed in this test. The microplate provided with the kit on which the results were measured was pre-coated with an antibody specific for serum AA. Standards or samples were then added to appropriate microplate wells with biotin-conjugated antibody specific for serum AA. Avidin conjugated with horseradish peroxidase was then added to each microplate well and incubated. Following the addition of tetra methyl benzidine substrate solution, color changes in the wells were determined according to the serum AA concentration in the sample. The enzyme-substrate reaction was terminated by adding sulfuric acid solution. Color change was measured using the ELISA method at a 450 nm + 10 nm wavelength on an ETI-MAX 3000 device (DiaSorin) at the Antalya Training and Research Hospital medical biochemistry laboratory. Serum AA concentrations in the samples were then determined by comparing the optical density of the samples with the standard curve.

A Cloud-Clone Corp. ELISA kit (catalog no. SEA885Hu 96 Tests; Cloud-Clone Corp., Wuhan, China) was used to measure serum AA levels. The assay range of the kit was 1.56-100 ng/mL, and its analytical sensitivity was 0.66 ng/mL. The results are expressed as ng/mL. The intra-assay coefficient of variability was < 10% and the inter-assay coefficient of variability was < 12% in 3 samples containing low, medium and high levels of serum AA. Standard curve concentrations used for ELISA assays were 100, 50, 25, 12.5, 6.25, 3.12, and 1.56 ng/mL.

### 2.2. Statistical analysis

Sample size calculation was performed using G Power software. The difference in serum AA levels, the primary outcome, between the groups was calculated at a minimum of 10%, with a power of 80% at a significance level of 5%, and at least 40 participants in each group. This difference of 10% derived from both the pilot study and our clinical trials.

General statistical analysis was performed on IBM statistics version 22 software. The Shapiro–Wilk test was applied to assess the normality of distribution of the study data. *P* > .05 was considered to represent normal distribution for these tests. Skewness and kurtosis values were checked, and normal Q-Q PLOT graphs were also used for non-normally distributed data. Normally distributed data were evaluated using Student *t* test, and non-normally distributed data using the Mann–Whitney *U* test. Pearson’s chi-square test was applied to compare categorical data. A receiver operating characteristic (ROC) curve was used to determine cutoff points with sensitivity and specificity values for predicting threatened miscarriage. *P* < .05 were regarded as statistically significant.

## 3. Results

**Figure 1.**
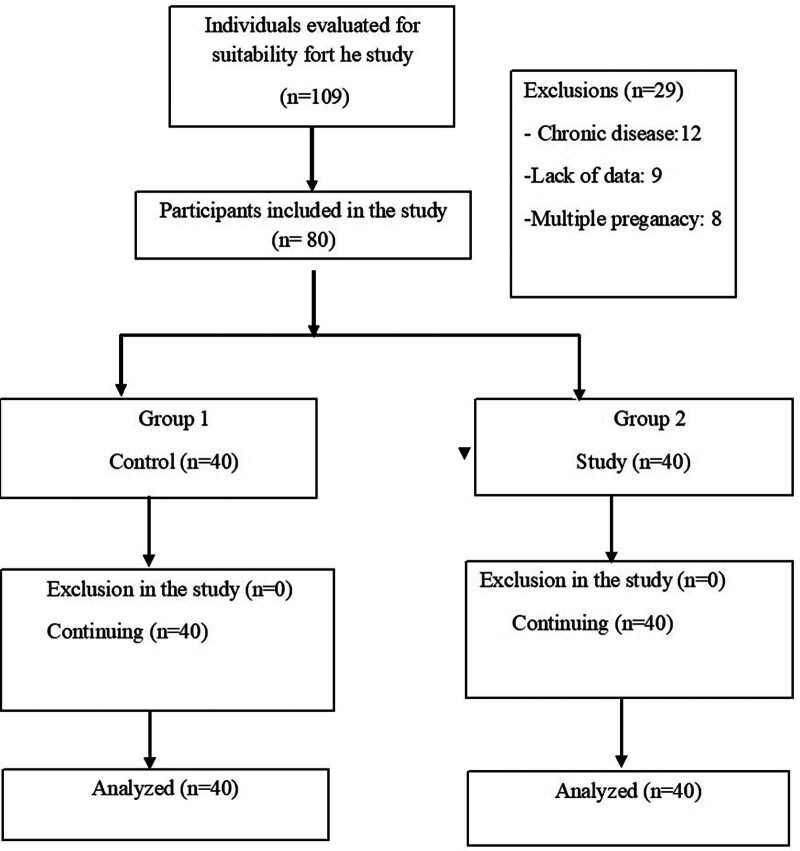
Study flowchart.

Eighty patients, 40 with HEG and 40 without active complaints, who presented to the Antalya Training and Research Hospital Gynecology and Obstetrics Emergency Department and gynecology and obstetrics clinics between July 01 and December 31, 2023, were included in the study (Fig. 1).

The sociodemographic structure and obstetric characteristics of the participants are shown in Table 1. No significant differences were determined between the HEG and non-HEG groups in terms of age (28.50 ± 5.66 vs 28.18 ± 4.98, respectively *P* = .641), BMI (25.33 ± 4.57 vs 24.40 ± 4.23, *P* = .331), education level (reader-not literate 0 [0%], primary education 7 [17.5%], high school 23 [57.5%], university 10 [25.0%] vs illiterate 0 [0%], primary education 8 [20.0%], high school 20 [50.0%], university 12 [30.0%], *P* = .795), economic level (low 29 [72.5%], middle 7 [17.5%], high 3 [7.5%] vs low 31 [20.0%], middle 13 [29%], 5, high 2 [5.0%], *P* = .795), place of residence (village 7 [17.5%], district 20 [50.0%], province 13 [32.5%] vs village 10 [25.0%], district 19 [47, 5%], province 11 [27.5%], *P* = .434), occupation (housewife 27 [67.5%], self-employed 6 [15.0%], clerical 7 [17.5%] vs housewife 29 [72.5%], self-employed 5 [12.5%], clerical 6 [15.0%]; *P* = .660), smoking (7 [17.5%] vs 10 [25.0%], *P* = .586], alcohol consumption [2 [5.0%] vs 4 [10.0%], *P* = .675), drug use (1 [2.5%] vs 3 [7.5%], *P* = .553], low [0 [0–0] vs 0 [0–0], *P* = .133), drug use (1 [2.5%] vs 3 [7.5%], *P* = .553), curettage (0 [0–0] vs 0 [0–0], *P* = .317), or gestational age (10.6 ± 2.5 vs 11.1 ± 2.1, *P* = .418). However, numbers of pregnancies (2 [1–3] vs 1 [0–1], *P* < .001), numbers of births (1 [0–1] vs 1 [0–1], *P* < .001), numbers of living children (1 [0–2] vs 1 [0–1], *P* < .001) and presenting complaints (nausea 0 [0%], nausea + vomiting 0 [0%], none 40 [100.0%] vs nausea 27 [67.5%], nausea + vomiting 13 [32.5%] [%], none 0 [%0]) differed significantly at the *P* < .001 level.

**Table 1:** The participants’ sociodemographic characteristics.

		Control (n = 40)	Study (n = 40)	*P*
Age (years)		28.75 ± 5.95	28.18 ± 4.98	.641
BMI (body mass index, kg/m^2^)		25.33 ± 4.57	24.40 ± 4.23	.334
Education level (n, %)	Illiterate	0 (0)	0 (0)	.795
	Primary	7 (17.5)	8 (2.0)	
	High	23 (57.5)	20 (5.0)	
	University	10 (25.0)	12 (3.0)	
Economic level (n, %)	Low	29 (72.5)	31 (2.0)	.846
	Intermediate	8 (20.0)	7 (17.5)	
	High	3 (7.5)	2 (5.0)	
Place of residence (n, %)	Village	7 (17.5)	10 (25.0)	.434
	Town	20 (50.0)	19 (47.5)	
	City	13 (32.5)	11 (27.5)	
Occupation	Housewife	27 (67.5)	29 (72.5)	.660
	Clerical worker	6 (15.0)	5 (12.5)	
	Manual worker	7 (17.5)	6 (15.0)	
Smoking (n, %)		7 (17.5)	10 (25.0)	.586
Alcohol consumption (n, %)		2 (5.0)	4 (10.0)	.675
Drug abuse (n, %)		1 (2.5)	3 (7.5)	.615
Gravity		2 (1-3)	1 (0-1)	**<.001** *
Parity		1 (0-2)	1 (0-1)	**<.001** *
Living child		1 (0-2)	1 (0-1)	**<.001** *
Miscarriage		0 (0-0)	0 (0-0)	.133
Dilatation-curettage		0 (0-0)	0 (0-0)	.317
Gestational age at admission (week)		10.6 ± 2.5	11.1 ± 2.1	.418
Complaint at admission (n, %)	Nausea	0 (0)	27 (67.5)	**<.001** *
	Nausea + vomiting	0 (0)	13 (32.5)	
	Absent	40 (100)	0 (0)	

* Statistically significant.

The participants’ laboratory results are given in Table 2. Significant differences were determined between the HEG and control groups in terms of serum hemoglobin (12.2 ± 0.8 vs 12.3 ± 0.9, respectively, *P* = .607), Htc (36.6 ± 2.9 vs 36.1 ± 4.9, *P* = .491), white blood cell (10.04 ± 3.74 vs 9.37 ± 2.73, *P* = .359), neutrophil (7.01 ± 3.32 vs 6.69 ± 2.51, *P* = .638), lymphocyte (2.27 ± 0.62 vs 2.05 ± 0.90, *P* = .211), and platelet (277.27 ± 57.30 vs 260.58 ± 54.85, *P* = .187) counts, NLR (3.27 ± 1.80 vs 3.99 ± 1.91, *P* = .188), PLR (128.29 ± 34.75 vs 157.38 ± 29.33, *P* = .173), TSH (1.16 ± 0.56 vs 1.81 ± 0.62, *P* = .004*), free T4 (0.93 ± 0.25 vs 0.87 ± 0.29, *P* = .578), albumin (41.69 ± 3.65 vs 41.65 ± 2.49, *P* = .948), alanine aminotransaminase (21.2 ± 9.9 vs 17.2 ± 8.6, *P* = .568), aspartate aminotransaminase (19.4 ± 5.1 vs 18.7 ± 6.1, *P* = .860), blood urea nitrogen (8.05 ± 2.57 vs 8.70 ± 3.38, *P* = 0, 0.336), creatinine (0.61 ± 0.09 vs 0.65 ± 0.13, *P* = .143), hs-CRP (6.80 ± 2.90 vs 8.27 ± 2.52, p: 0.346), sodium (138.05 ± 3.15 vs 138.70 ± 4.47, *P* = .411), potassium (4.1 ± 0.7 vs 3.8 ± 0.2, p: 0.346), serum potassium (3.9 ± 0.3 vs 3.8 ± 0.2, *P* = .001*), and AA levels (7.29 ± 2.61 vs 10.74 ± 3.04, *P* < .001*).

**Table 2:** The participants’ laboratory values

	Control (Group 1) (n = 40)	Study (Group 2) (n = 40)	*P*
Hb (g/dL)	12.2 ± 0.8	12.3 ± 0.9	.607
Htc (%)	36.6 ± 2.9	36.1 ± 4.9	.491
WBC (10^3^/mm^3^)	10.04 ± 3.74	9.37 ± 2.73	.359
Neutrophil (10^3^/mm^3^)	7.01 ± 3.32	6.69 ± 2.51	.638
Lymphocyte (10^3^/mm^3^)	2.27 ± 0.62	2.05 ± 0.90	.211
Platelet (10^3^/mm^3^)	277.27 ± 57.30	260.58 ± 54.85	.187
NLR	3.27 ± 1.80	3.99 ± 1.91	.188
PLR	128.29 ± 34.75	157.38 ± 29.33	.173
TSH (uIU/mL)	1.81 ± 0.62	1.16 ± 0.56	**.004** *
Free T4 (ng/dL)	0.93 ± 0.25	0.87 ± 0.29	.578
Albumin (g/L)	41.69 ± 3.65	41.65 ± 2.49	.948
ALT (U/L)	21.2 ± 9.9	17.2 ± 8.6	.568
AST (U/L)	19.4 ± 5.1	18.7 ± 6.1	.860
BUN (mg/dL)	8.05 ± 2.57	8.70 ± 3.38	.336
Creatinine (mg/dL)	0.61 ± 0.09	0.65 ± 0.13	.143
hs-CRP (mg/L)	6.80 ± 2.90	8.27 ± 2.52	.346
Sodium (mmol/L)	138.05 ± 3.15	138.70 ± 4.47	.411
Potassium (mmol/L)	4.1 ± 0.7	3.8 ± 0.2	**.001** *
Amyloid-A (ng/mL	7.29 ± 2.61	10.74 ± 3.04	**<.001** *

ALT = alanine aminotransferase, AST = aspartate aminotransferase, BUN = blood urea nitrogen, CRP = C-reactive protein, Hb = hemoglobin, Htc = hematocrit, NLR = neutrophil-to-lymphocyte ratio, PLR = platelet-to-lymphocyte ratio, T4 = thyroxine, TSH = thyroid-stimulating hormone, WBC = white blood cell.

* Statistically significant.

**Figure 2.**
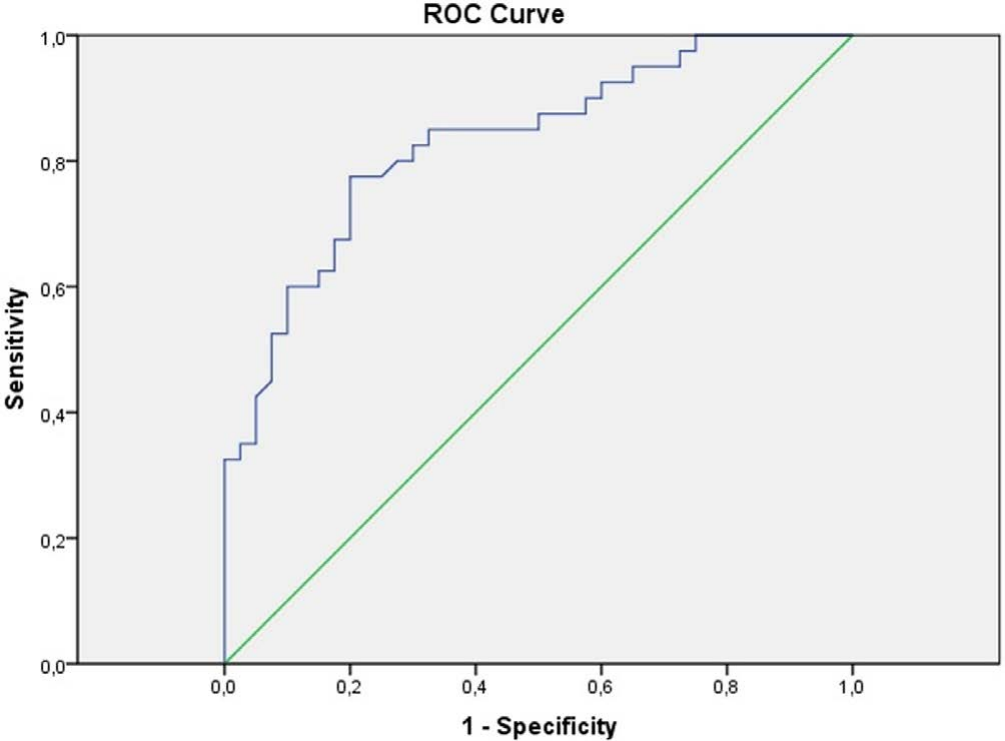
ROC curve for serum amyloid A. ROC = receiver operating characteristic.

At ROC analysis, the area under the curve (Fig. 2) (AUC: 0.881) was statistically significant for serum AA (*P* < .001), with a cutoff value of ≥ 8.79 (95% confidence interval [CI] 0.743–0.919, sensitivity 87%, specificity 80.2%). The positive and negative predictive values of serum AA were 81.1% and 80.4%, respectively (Table 3).

**Table 3:** Sensitivity, specificity, positive predictive value, and negative predictive values for amyloid A.

	AUC	*P*	Lower limit	Upper limit	Sensitivity	Specificity	PPV	c
Amyloid A	0.881	<.001	0.743	0.919	87.4%	80.2%	81.1%	80.4%

AUC = area under the curve, NPV = negative predictive value, PPV = positive predictive value.

## 4. Discussion

This is the first study compared the blood parameters and serum AA levels of women with HEG and healthy pregnant women with no complaints in order to detect the presence of inflammation, thought to play an important role in the pathophysiology of HEG. While there was no statistically significant differences in sociodemographic characteristics, obstetric data, or inflammatory blood parameters were determined between the groups, serum AA levels were significantly higher in the HEG group.

HEG is a very common obstetric emergency, the etiopathogenesis of which is still not fully understood. Possible causes include an extraordinary increase in beta-hCG, high estrogen and progesterone levels, hyperthyroidism, high prostaglandin E2, and Helicobacter pylori infection. Nausea and vomiting in pregnant women are also severe when B-HCG levels are highest.^[14,15]^ Estrogen level elevation in pregnant women has a direct effect on the central nervous system and a slowing effect on gastric emptying, and progesterone delays gastric emptying by reducing smooth muscle contractions and motility.^[16,17]^

Studies have shown that increased hCG in pregnant women causes temporary hyperthyroidism due to its similarity to thyroid-stimulating hormone (TSH), and this exacerbates nausea and vomiting during pregnancy. A strong relationship has been shown between H. pylori infection, which causes gastric and duodenal ulcers, and HEG in recent years.^[14]^

HEG may result in adverse perinatal outcomes such as abortion, low birth weight, prematurity, birth of a baby with a low APGAR score, preterm labor, preeclampsia, fetal growth restriction, and placental abruption in the later stages of pregnancy.^[18,19]^ These adverse perinatal outcomes increase both fetal morbidity and mortality. Medical treatment that can be applied to prevent these adverse perinatal outcomes resulting from HEG will reduce both maternal and fetal morbidity. Costs will also decrease with early treatment, thus benefitting both the national economy and hospitals.

There is no specific diagnostic test for predicting the prognosis of HEG, and due to the limited numbers of the studies on maternal serum markers, we think that this research will make a particularly useful contribution to the subject.

Treatment in HEG includes reducing symptoms, improving the patient’s quality of life, correcting hypovolemia, ketoacidosis and electrolyte abnormalities, and preventing complications such as vitamin deficiency and electrolyte irregularities that may occur due to vomiting.

In terms of BMI, some studies have observed no difference between patients with HEG and a control group,^[20]^ while others have reported lower BMI in patients with HEG.^[21]^ No significant difference was determined between the groups in the present study.

From the perspective of gestational age, HEG is reported to be more common in first pregnancies,^[22]^ and was observed significantly more frequently in first pregnancies in the present study, a finding consistent with the literature.

Previous research has reported a negative relationship between HEG and TSH, and that increased B-HCG in HEG suppresses TSH while increasing thyroid hormones with a TSH-like effect.^[23]^ In the present study, while TSH levels were significantly lower in the patients with HEG than in the control group, no significant difference was determined in terms of free thyroid hormones.

Both NLR and PLR, 2 inflammatory biomarkers, increase in preeclampsia, cholestasis of pregnancy, ovarian, breast, and colorectal cancer, coronary artery diseases, ulcerative colitis, and pancreatitis.^[24]^ They have also been identified as important prognostic markers in determining average life expectancy in cases of primary tubal uterine cancer and are associated with lymph node involvement in vulvar squamous cell carcinoma.^[25]^ Researchers have suggested that NLR exhibits 78% sensitivity and 80% specificity, and PLR 100% sensitivity and 43% specificity, in the preoperative diagnosis of acute appendicitis in pregnant women, and that these may be effective biomarkers in predicting the prognosis of acute pancreatitis.^[24]^ In addition, studies have shown maternal serum NLR and PLR values increase in pregnant women with abortus imminens and abortion compared to healthy pregnant women.^[26]^

Serum AA is an important acute phase reactant that rises in response to infection and inflammation. No previous studies have investigated serum AA levels in pregnant women with HEG, and the present research is original from that perspective. Serum AA levels have been shown to rise in colorectal carcinoma, ovarian cancer, uterine carcinomas and some brain tumors, some inflammatory bowel diseases, and polycystic ovary syndrome, conditions in which inflammation may be involved in the etiopathogenesis, and maternal serum AA levels also rise in preterm births due to chorioamnionitis.^[12,27]^

In the present study, serum AA levels in women with HEG were significantly higher than in the healthy pregnant women. ROC analysis revealed a diagnostic value in predicting HEG of 8.79 (AUC: 0.881, CI: 0.743–0.919, sensitivity: 0.874, specificity: 0.802, positive predictive value: 81.1, negative predictive value: 80.4).

A particular strength of this research is that it was performed as a prospective cohort study, homogenization being achieved in terms of sociodemographic and obstetric data, and the bias rate being low. Another strength is that since the cases represent the central part of Turkey, the results can be adapted to the entire country. However, a potential limitation is that the study was conducted in a single tertiary reference center.

In conclusion, this is the first study investigated serum inflammatory biomarkers and serum AA levels in pregnant women with HEG. While no statistically significant differences were found in terms of hs-CRP, NLR, or PLR values, serum AA levels were significantly higher in the HEG group. This shows that inflammation plays a role in the etiopathogenesis of HEG and that maternal serum AA levels can be used as a biomarker in cases of HEG. Further prospective studies involving larger numbers of participants are now required to confirm the accuracy of our results.

## Acknowledgments

The authors thank Dr Hasan Ali Inal for his support in valuable comments.

## Author contributions

**Investigation:** Durmus Onder, Meryem Busra Birsen, Derya Erturk, Ahmet Ilker Eryilmaz, Ozgur Ozdemir, Guzin Aykal, Zeynep Ozturk Inal.

**Methodology:** Durmus Onder, Meryem Busra Birsen, Derya Erturk, Ahmet Ilker Eryilmaz, Guzin Aykal.

**Resources:** Durmus Onder.

**Supervision:** Durmus Onder, Meryem Busra Birsen, Derya Erturk, Ahmet Ilker Eryilmaz, Ozgur Ozdemir, Guzin Aykal, Zeynep Ozturk Inal.

**Writing – original draft:** Durmus Onder, Zeynep Ozturk Inal.

**Writing – review & editing:** Durmus Onder, Zeynep Ozturk Inal.
